# Perception of a Black Room Seen Through a Veiling Luminance

**DOI:** 10.1177/2041669520973698

**Published:** 2020-11-18

**Authors:** Alan Gilchrist, Michael S. Langer

**Affiliations:** Rutgers University, Newark, New Jersey, United States; McGill University, Montreal, Quebec, Canada

**Keywords:** black room, veiling luminance, lightness, mutual illumination, image contrast, luminance gradients

## Abstract

When a black room (a room painted black and filled with objects painted black) is viewed through a veiling luminance, how does it appear? Prior work on black rooms and white rooms suggests the room will appear white because mutual illumination in the high-reflectance white room lowers image contrast, and the veil also lowers image contrast. Other work reporting high lightness constancy for three-dimensional scenes viewed through a veil suggests the veil will not make the room appear lighter. Because mutual illumination also modifies the pattern of luminance gradients across the room while the veil does not, we were able to tease apart local luminance gradients from overall luminance contrast by presenting observers with a black room viewed through a veiling luminance. The room appeared white, and no veil was perceived. This suggests that lightness judgments in a room of one reflectance depend on overall luminance contrast only.

In the work reported here, we addressed a very simple question: Would a black room seen through a veiling luminance appear as a white room in plain view or as a room darker in color than white, behind a veiling luminance? Two prior lines of work suggest opposite answers to this question. First, a summary of that work, involving black rooms and veiling luminance, is presented. 

## Research on Black Rooms

**Figure 1.**
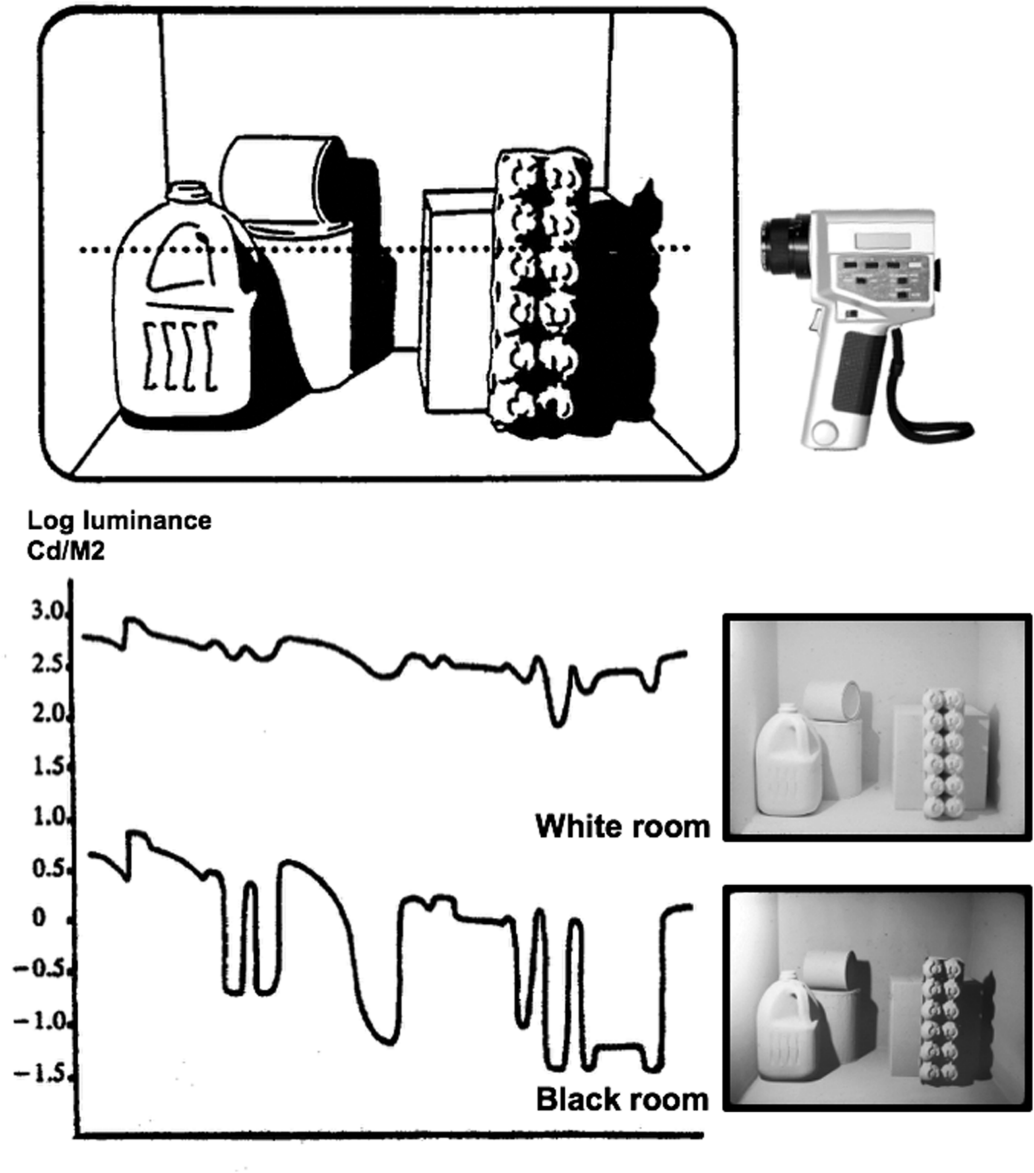
Top: Sketch of room showing path of closely spaced photometer readings, with eight target locations. Bottom: Luminance profiles based on photometer readings from the actual rooms. Images in lower right are photographs, not computer graphics. Note that the black room appears lighter in the photograph than it does when viewed directly. Adapted from Gilchrist and Jacobsen (1984).

In work by Gilchrist and Jacobsen (1984), subjects looked into one of two miniature rooms, each filled with simple geometric objects, as seen in Figure 1. In one room, every object and every wall was painted completely matte black. In a second room, everything was painted matte white. The subjects matched lightness values and illumination intensities at eight target locations in the room.

That work was inspired by a puzzling question concerning the perception of lightness in a world in which every surface has the same reflectance (shade of gray). Would lightness be veridical without the possibility of comparing different shades of gray? And, apart from absolute luminance, would an all-black world appear different from an all-white world? Intuitively, the answer was yes, but what information could be used to make the distinction?

The actual results showed that the white room appeared completely white, while the black room appeared as a middle gray. Those results changed little even when the overall illumination was raised in the black room and lowered in the white room such that the luminance at every point in the black room was higher than the corresponding point in the white room.

Analysis of the structure of light showed that the different appearance of the rooms was due to mutual illumination (Ruppertsberg & Bloj, 2007), which refers to reflected light from one surface that illuminates a neighboring surface. In a white room, every surface reflects about 90% of the light it receives. Thus, there is mutual illumination everywhere and, as a result, the retinal image produced is quite homogeneous (low contrast). In a black room, there is very little mutual illumination and, as a result, the image of the black room shows very high contrast. Perhaps luminance amplitude would be a better term, but until the critical feature of the black room image is determined empirically, we will use the more general term contrast.

A further goal of the work was to demonstrate that illuminance edges (caused by a change in illumination intensity) are discriminated from reflectance edges (caused by a change in pigment). This was a time when dramatic findings from experiments on stabilized images (Yarbus, 1967) and Craik–O'Brien contours (Craik, 1966; O'Brien, 1958) drove an increasing recognition of the importance of edges. Even a rough veridicality of lightness was not possible without the visual discrimination of illuminance edges from reflectance edges, yet the distinction was rarely made. Each of the rooms contained many visible edges. But these were all illuminance edges, given that any reflectance edges were eliminated by painting everything with a single shade. Clearly illumination edges are visible. They are not so shallow that they fall below threshold, as suggested by Land and McCann (1971) and others. But are they correctly perceived as illuminance edges?

In each room, reflectance matches made by observers at the eight target locations were approximately equal, while the matched illuminance values at those same locations varied roughly with actual illuminance values. This showed that the edges were correctly seen as illuminance edges.

Returning to our current experiment, if the distinction between black rooms and white rooms depends on the degree of contrast in the image, then we would predict that a black room seen through a veiling luminance of sufficient brightness would appear as a white room, given that the veil reduces image contrast.

## Research on Veiling Luminance

On the other hand, work on veiling luminance suggested a different outcome. A veiling luminance is a homogeneous layer of light that is added to the image. For example, perception can be hampered when looking into a store window or a car windshield because light from the sky reflected off the front of the glass adds a layer of light, which, like fog, reduces image contrast. Slides projected onto a screen lose contrast when the room lights are on because ambient light reflecting off the screen constitutes a veiling luminance. Early studies found substantial changes in the lightness of simple stimuli, such as a disk/ring pattern, when they are covered by a veiling luminance (Fry & Alpern, 1954; Rushton & Gubisch, 1966). Consistent with this, Gilchrist and Jacobsen (1983) found that a two-dimensional Mondrian pattern with patches spanning the entire (30:1) range from white to black appeared to contain only light grays (Munsell 9.5 to 7) when seen through a veil that reduced the range to about 2:1. However, they obtained almost 100% constancy when the same veil covered a complex, three-dimensional display with the same luminance range.

This finding of constancy in a three-dimensional display covered by a veil, but not in a two-dimensional display, implies that that a black room, being three-dimensional and covered by a veil, will evoke the perception of a gray room (similar to its appearance without a veil) with a separate veil in front of it.

There is a further argument that can be brought to bear on our question. In the work on black rooms and white rooms, the high image contrast produced by the black room was reduced by painting the room white, whereas in the veiling luminance work, image contrast was reduced by adding a veil of light. These two methods are not equivalent, although it can be said that both methods reduce contrast. The light that is added to the image by painting the room white is not a homogeneous layer of light.

**Figure 2.**
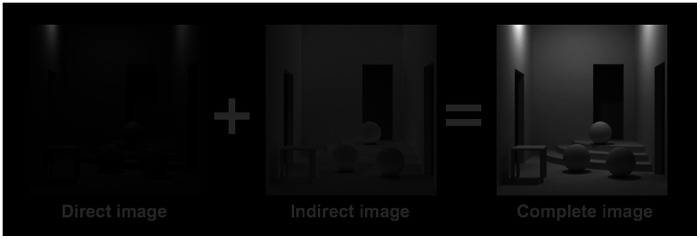
Right: Simulated image of a white room containing objects. Left: Image composed solely of one-bounce light rays (no indirect illumination). Middle: Image composed of all light rays except those in the direct image.

It is possible to visualize the mutual illumination in the white room using computer graphics (Larson & Shakespeare, 1998). Figure 2 shows a white scene (reflectance 0.9) that was rendered twice under the same lighting conditions, once without mutual illumination (left) and once with mutual illumination (right). The difference of these two images (middle) is the mutual illumination component on its own.

The direct image is roughly equal to the image produced by a black room under proportionally stronger illumination. As can be seen in these images, adding the indirect image is different than adding a homogeneous veil of light, because the indirect image is not the same as a homogeneous sheet of light. The experiments reported here serve as an additional test of whether such differences are important to the visual system, or whether the important issue is merely the gross level of contrast in the image.

## Method

### Apparatus

**Figure 3.**
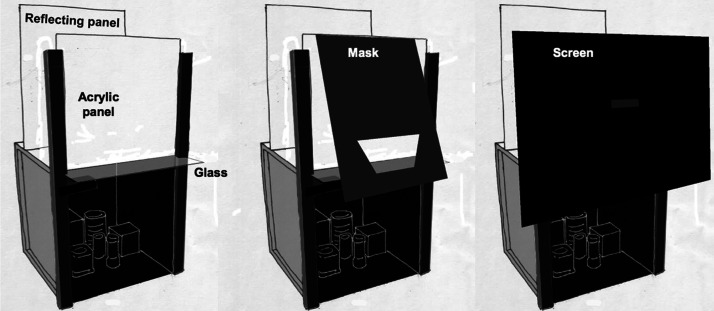
Left: Room was seen only through protruding sheet of clear glass that reflected light transmitted through acrylic panel. Two 15-watt florescent tubes illuminated a panel that reflected light onto acrylic panel. Middle: A tilted mask occluded everything except the room interior visible through trapezoidal aperture. Right: Viewpoint was limited by horizontal slot in screen that covered the front of the apparatus.

The apparatus, shown in Figure 3, consisted of a small, approximately cubic room (37 cm wide, 40 cm high, and 42 cm deep), attached to a wall with its bottom 89 cm above the floor. The interior was painted entirely of matte black (4.6%) and filled with six objects, also painted black. Illumination was provided by a 35-watt quartz halogen bulb mounted in the near upper left-hand corner, out of the observer’s visual field. The veil was created by reflecting light off of a sheet of clear glass that extended 10 cm horizontally from the top front of the room. The reflected light came from a 33 by 24 cm panel of translucent acrylic mounted in a vertical position flush with the front of the room. This panel was homogeneously illuminated by light reflected from a large opaque white panel located 16 cm behind the acrylic panel and parallel to it. The opaque panel was illuminated by two 15-watt fluorescent tubes mounted vertically, immediately behind the left and right edges of the acrylic panel. Observers viewed the room by looking downward through a horizontal rectangular aperture (3.8 by 8.9 cm) in a large black foam-board panel. The observer’s field of view was limited to the room itself by means of a trapezoidal aperture in the center of another black foam-board mask mounted in a slanted position between the viewing aperture and the reflecting glass. In the no-veil condition, a black baffle (37 cm × 31 cm) was inserted in front of the acrylic panel to block its reflection onto the glass.

**Figure 4.**
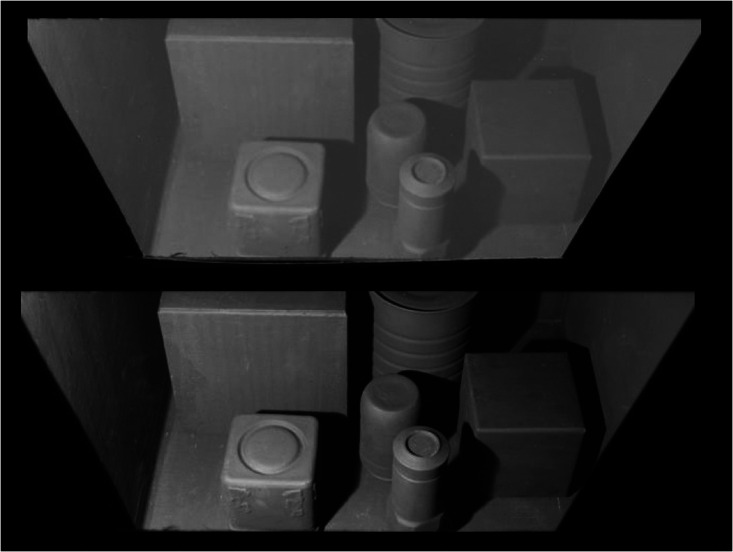
Photographs of black room with veil (upper) and without veil (lower). The true percept of the rooms is not portrayed by the photographs.

The highest luminance in the room (without the veil) was 5.3 cd/m^2^, and the luminance range was approximately 30:1. Figure 4 shows the room with and without the veil. Because the luminance of the veil was 5.5 cd/m^2^, the veil reduced the luminance range to approximately 2:1. Matching was done using a 16-step Munsell chart, separately illuminated and housed in a metal chamber mounted 45 cm directly below the viewing slot. The luminance of the white chip was 360 cd/m^2^

Note that, due to nonlinearities in the image capture process and the absence of motion parallax, stereo, and other cues, viewing the photographs in Figures 1 and 4 cannot replicate the visual experience of viewing the rooms themselves.

### Subjects

A total of 30 subjects volunteered to participate to satisfy a course requirement. They were naïve with respect to the purpose of the experiment. One group of 15 subjects viewed the black room through the veil, and a separate group of 15 viewed the room without the veil.

### Procedure

The subject was asked to look in a downward direction through the slot in the screen and report what they saw in words. Then, he or she was asked to match the gray shade of the surfaces in the room by selecting a matching chip from the Munsell chart. Subjects were then asked whether it appeared that they were looking through any kind of fog or reflection. Dark adaptation was not used, given that pilot work showed it to have no effect on lightness.

All data analyses were conducted by first transforming the Munsell matches into log reflectance values.

## Results

**Figure 5.**
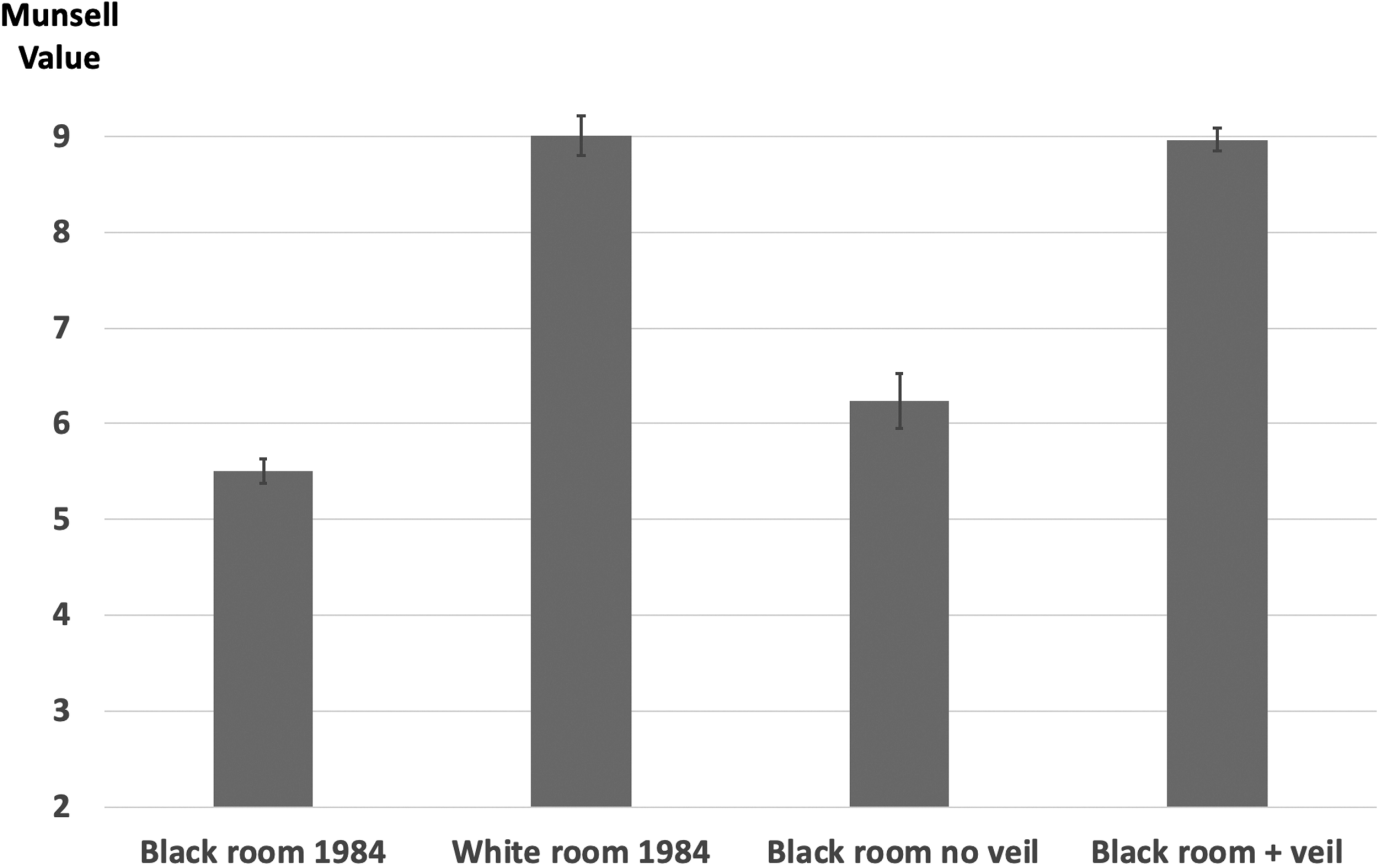
Mean Munsell matches for black rooms and white rooms in original study (left) and for black room with and without veil present in current study (right).

All of the subjects in the Gilchrist and Jacobsen experiment reported that all the surfaces in the room were painted with the same shade of paint, and informal comments by our subjects were consistent with this. This is equivalent to saying that all of the gradients and step changes were perceived as changes in illuminance, not pigment. As for the specific lightness seen in the room, Figure 5 shows the results for the black room with and without the veil, along with the results for the black room and white room reported by Gilchrist and Jacobsen (1984). The black room seen through the veil was perceived as white, with an average Munsell value of 9.0. This value is almost identical to the average value reported in 1984 for the white room. When asked whether it appeared that they were looking through a fog or a reflection, all 15 subjects said no. The average value reported for the black room without the veil was 6.2, slightly higher than the value of 5.5 reported in 1984. We believe the reason for this difference is the limited view of the room in our current setup. Indeed, in an earlier pilot study with an even smaller field of view, the room was matched to a Munsell 6.9. In the 1984 study, the room filled most of the observer’s field of view.

## Discussion

These results provide a clear answer to our original question. A black room viewed through a veiling luminance (without visible borders) appears to be a white room. Thus, the critical factor seems to be the gross level of contrast, or luminance amplitude, in the room. The specific function of the luminance gradients does not seem to be important.

As suggested earlier, the retinal image produced by a black room seen through a veiling luminance is not the same as the image produced by a white room. This can be understood by referring to Figure 2. The image of the black room is very similar to what we have called the direct image; that is, the high-contrast image made by direct lighting only, excluding any contribution by mutual illumination (that is, the indirect image). We can reduce the image contrast either by painting the black room white or by adding a veiling luminance. But while adding a veiling luminance adds a homogeneous layer of light, painting the room does not—it adds the indirect image, shown in Figure 2, which is, of course, not homogeneous. Thus, the luminance gradients in a white room are different from those in a black room plus veil. (The rooms are the same only in their gross level of reduced contrast.) But this difference did not impact the lightness judgments.

This finding is consistent with other results showing a visual indifference to the specific form of a gradient. Arend and Goldstein (1990) simulated the projection of different illumination gradients across a Mondrian pattern and measured the degree of lightness constancy for targets at the high and low ends of the gradient. They found that the amount of constancy did not depend on the shape of the gradient (linear, accelerating, or step function) but merely on the gross luminance change over the gradient.

However, these results appear to contradict those of Gilchrist and Jacobsen (1983) who, while finding no constancy for a Mondrian pattern seen through a veil (to no surprise), found almost 100% constancy for a three-dimensional still life seen through a veil. However, that three-dimensional display contained objects of different color and different shades of gray, neither of which were present in our black room study. Why would that make a difference?

Different colors and different shades, when covered by a veiling luminance, produce correlations in the image that signal the presence and strength of a veiling luminance. When, for example, a vertical colored cylinder is lit (with neutral light) from one side and viewed through a neutral veiling luminance, the gradient across the cylinder exhibits a positive correlation between luminance and saturation. The high luminance end is more saturated than the low luminance end because the high end is composed of a mixture of the veil plus a lot of colored light from the cylinder, while the low end has a mixture of the veil plus much less colored light from the cylinder.

Likewise, when shadow edges fall on coplanar objects of different shades of gray, there is a correlation between the luminance of the object and the contrast at the edge of the shadow. Shadow edges on a black object will show low contrast (the edge is swamped by the veil), perhaps below threshold, while shadow edges on a white object retain much of their contrast (because the veil is weaker relative to the white object). A variant of this occurs when the edge of a shadow falls across the boundary between two neighboring coplanar surfaces that differ in reflectance. This creates a ratio-invariant X junction (Gilchrist et al., 1983). When no veil is present, then the luminance ratio (a measure of local contrast) at the reflectance edge is the same on both sides of the illumination edge (and equal to the ratio of reflectances). However, when a veil is present, then the reflectance edge under the lower illumination level will have a lower contrast across it than the part of the edge under higher illumination, as the veil is additive (unlike higher illumination, which is multiplicative). This luminance-contrast correlation applies to the illumination boundary itself, as well. The shadow edge on the higher reflectance shows a higher contrast than that edge on the lower reflectance.

Luminance-saturation gradient correlations and luminance-contrast correlations exist only when a veiling luminance is present. But in our black room experiment, even though a veil is present, these correlations are not present because our objects all have the same shade of gray and there are no colored objects.

However, some correlations remain. Surfaces with different levels of illumination can behave like surfaces of different reflectance. Thus, a luminance-contrast correlation exists between the intensity of illumination on a surface and the contrast at the edge of a shadow falling on it. In Figure 4, notice that the edge of the shadow that falls on the more weakly lit right-hand wall of the room has a lower contrast than the edge of the shadow that falls on the more brightly lit large cube to the upper left.

In principle, deep concavities of the scene, whose geometry can be perceived based on many available three-dimensional cues, provide information that a veil is present, even in a scene of one reflectance. Deep concavities tend to be dark in both the direct and indirect image (Langer, 1999). An example is the concavities in the egg carton in Figure 1. When a veil is added to this scene, the luminance of this deep concavity is raised by the veil (along with the luminance of other surfaces) above the near-zero level of the tunnel through which the scene is viewed.

But apparently these residual products of our veil were unable to compensate for the absence of the other correlations. In this case, the visual system fails to disentangle the two possible causes of the low contrast, namely (a) a veil is present and the room is black, or (b) no veil is present and the room is white. Observers stated that they did not perceive a veil, and their reflectance judgments are consistent with the no-veil perception. Simply put, the black rooms under a veil had low contrast, similar to white rooms under no veil, and so the black rooms under a veil appeared white.

The theoretical implications of our findings are not clear. The luminance-contrast and luminance-saturation correlations that seem to allow discounting of veiling luminance are textbook examples of Gibson’s higher order variables. But the fact that a black room can be made to look like a white room by covering it with a veiling luminance, even though this only crudely replicates the distribution of light in a white room, appears to support midlevel approaches to lightness. But overall it seems safe to say that there is currently no theory of lightness that can accommodate our findings.
